# Repercussions of the sexual experiences of women with a history of domestic violence: A Grounded Theory

**DOI:** 10.1590/0034-7167-2024-0561

**Published:** 2026-10-19

**Authors:** Luana Moura Campos, Nadirlene Pereira Gomes, Lilian Conceição Guimarães de Almeida, Jordana Brock Carneiro, Josinete Gonçalves dos Santos, Janli Kelly Pereira Fontes dos Santos, Joyce Mendes Paim, Rebeca dos Santos Santos

**Affiliations:** I Universidade Estadual do Sudoeste da Bahia. Jequié, Bahia, Brazil; II Universidade Federal da Bahia. Salvador, Bahia, Brazil; III Universidade do Estado do Rio de Janeiro. Rio de Janeiro, Rio de Janeiro, Brazil; IV Hospital Sofia Feldman. Belo Horizonte, Minas Gerais, Brazil

**Keywords:** Sexuality, Intimate Partner Violence, Gender Analysis, Women’s Health, Grounded Theory., Sexualidad, Violencia de Pareja Íntima, Análisis de Género, Salud de la Mujer, Teoría Fundamentada.

## Abstract

**Objectives:**

to understand the repercussions of sexuality experiences on the lives and health of women with a history of intimate partner violence.

**Methods:**

a qualitative study grounded in Grounded Theory and the theoretical framework of gender and patriarchy as proposed by Kate Millet. Seventeen women assisted by the Ronda Maria da Penha operation and ten Family Health Strategy professionals in Salvador, Bahia, were interviewed. This study brings together data from the component Consequences of the Paradigmatic Model.

**Results:**

the following categories emerged: Experiencing repercussions on physical health, Experiencing repercussions on psychological health, Experiencing repercussions on the relationship, and Experiencing the discovery of sexual pleasure.

**Final Considerations:**

sexuality experiences were marked by physical and psychological repercussions, such as pain during sexual intercourse, sexually transmitted infections, and psychological distress, as well as feelings of discomfort, repulsion, and blockage toward sexual relations. Additionally, some women experienced the discovery of pleasure in sexual relations.

## RESUMEN

**Objetivos:**

conocer las repercusiones de las experiencias de la sexualidad en la vida y la salud de mujeres con antecedentes de violencia conyugal.

**Métodos:**

investigación cualitativa basada en la *Grounded Theory* y en el marco teórico de género y patriarcado desarrollado por Kate Millet. Se entrevistó a 17 mujeres atendidas por la Operación Ronda Maria da Penha y a diez profesionales de salud de la Estrategia de Salud de la Familia, en Salvador, Bahia. Este estudio reúne datos del componente Consecuencias del Modelo Paradigmático.

**Resultados:**

se identificaron las siguientes categorías: Experimentando repercusiones en la salud física, Experimentando repercusiones en la salud psicológica, Experimentando repercusiones en la relación y Experimentando el descubrimiento del placer sexual.

**Consideraciones Finales:**

las experiencias de la sexualidad estuvieron atravesadas por repercusiones físicas y psicológicas, como dolor durante el acto sexual, infecciones de transmisión sexual y sufrimiento psíquico, además de sensaciones de incomodidad, repulsión y bloqueo hacia las relaciones sexuales. Asimismo, algunas mujeres experimentaron el descubrimiento del placer en la relación sexual.

## INTRODUCTION

Sexuality is related to the way individuals live in the world, their interactions with others, their desires, and their sexual satisfaction^(1)^. This experience is influenced by several factors, such as education, culture, and patriarchal values, and it can affect the health of men and women differently due to gender inequalities^(2)^.

The social construct of gender shapes the predictable ways in which boys and girls relate to the world, to affection, emotions, pleasure, and consequently to sexuality. Within an unequal gender culture, boys from an early age are guided toward practices of sexual satisfaction, the prioritization of personal desires, and the behavioral standards of hegemonic masculinity. In contrast, for most girls and women, opportunities for learning and experiences related to sexuality remain limited, restricting them to providing pleasure rather than experiencing pleasure themselves^(3-5)^. This dichotomy is reinforced by Schweiger^(5)^ in his study on gender socialization and sex education, which highlights the need for a gender-sensitive approach to sexual education - one that challenges hegemonic masculine norms and promotes alternative ways of experiencing sexuality and affective relationships^(5)^.

For women, the benefits arising from experiences permeated by fantasies, touch, and desire are taken away, and their relationship with the other is reduced to the purpose of reproduction or to the man’s sexual satisfaction - an individual who is socially positioned as having exclusive sexual ownership over the woman, without reciprocity^(3,6)^. Thus, those who recognize sexual practices, behaviors, and exchanges as something that meets their own intimate needs, and who disregard social sanctions, are judged as sinful or vulgar^(7)^. This social behavior represses and coerces women not to explore their sexuality or to experience possibilities of relating to themselves and to others^(8)^.

It is from these deeply rooted beliefs that intimate relationships are shaped, in which women and men reproduce learned behavioral patterns. Within this dynamic, marital relationships may emerge in which repression and sanctions occur in an exacerbated manner, marked by disrespect and violence. Patriarchy reinforces these masculine characteristics, grounded in radical, sudden, and violent behaviors^(3)^. Such relationships are generally characterized by restrictions and by a sense of male ownership over women’s bodies^(3)^, becoming fertile ground for negative experiences in the realm of sexuality^(7,9)^, with potential impacts on life and health.

Given the numerous constraints placed on women’s sexuality, within conjugal relationships, the harms resulting from a sexist society and violent partnerships can lead, throughout life, to situations such as depression, low self-esteem, social isolation, among others^(10)^. It is important to highlight the covert nature of violence in the realm of sexuality, as many women experience its repercussions in silence-often associated with feelings of shame or fear of social judgment, especially when such violence occurs within intimate relationships^(11)^.

Therefore, it is essential to consider the role of health professionals, particularly nursing, in identifying the repercussions of violence related to sexuality, in order to build pathways of support for women who experience such harm. The originality of this study lies in its effort to understand the phenomenon of sexuality experiences among women who have lived through conjugal violence, with the aim of informing strategies to address this issue in sexual and reproductive health care. In this context, there is a clear need to recognize signs that can guide health professionals in improving women’s quality of life in the domain of sexuality.

### Study relevance

This article is derived from the doctoral dissertation entitled Sexuality of women with a history of conjugal violence: understanding the phenomenon from a Grounded Theory^(12)^ deposited in the institutional repository: https://repositorio.ufba.br/bitstream/ri/38543/1/TESE%20-%20LUANA%20MOURA%20CAMPOS.pdf. The research data are available in a repository.

## OBJECTIVES

To understand the repercussions of sexuality experiences on the lives and health of women with a history of intimate partner violence.

## METHODS

### Ethical aspects

The study was approved by the Research Ethics Committee of the School of Nursing at *Universidade Federal da Bahia (“*UFBA”, Federal U88960217.6.0000.5531 and opinion no. 2.639.224/2018 and CAAE: 88960217.6.0000.5531 and complied with Resolutions 466/2012 and 510/2016 of the *Conselho Nacional de Saúde* (National Health Council). To preserve participants’ identities, the women interviewed were identified by a code composed of the letter *M* (referring to “mulher”/“woman”) followed by the interview order number, for example, “M1”. In the case of health professionals, the coding combined the letter corresponding to the professional category (nurse-E, physician-ME, and psychologist-P) with the number indicating the interview order. It is important to note that the Informed Consent Form was obtained from all participants through a printed document. Before signing, the information contained in the form - such as the study objectives, the purpose of the research, and the procedures involved - was read aloud to the participants.

### Theoretical-Methodological Framework

The data were analyzed using Grounded Theory (GT), specifically the updated Straussian strand^(13)^, and based on the theoretical assumptions of gender and patriarchy from the perspective of Kate Millet (1970)^(3)^. This framework developed feminist discussions on gender through what we now refer to as social markers, encompassing relations involving race, caste, class, and sex. It also drew attention to the way it approaches “sex”, considering it a category subject to political influence and therefore a central axis that consolidates the patriarchal system as an institution of control^(3)^.

### Type of study

This is a qualitative study linked to the main project “Addressing conjugal violence within the Unified Health System: a social technology involving women, men, and Primary Care professionals”, submitted to the Research Program for the *“SUS”* (Unified Health System): Shared Health Management - PPSUS/BA call for proposals. To guide the reporting of the qualitative research, the Consolidated Criteria for Reporting Qualitative Research (COREQ) checklist was used. The data collection team consisted of six of the eight authors of the study, all women with different levels of academic training, including undergraduate students, master’s students, and PhD-level nurses. All had prior experience conducting qualitative interviews and worked as nurses and scientific initiation fellows. This previous experience provided greater sensitivity when addressing intimate and delicate issues related to the study’s topic.

### Theoretical assumption

Women with a history of intimate partner violence receive sexual education rooted in structural machismo and experience their sexuality in a repressed and non pleasurable way, centering it on the spouse’s satisfaction. This compromises their physical and mental integrity and leads them to seek professionals working in the Family Health Strategy (FHS). This situation highlighted the need to investigate a second sample group composed of these professionals.

### Study setting

The present study was conducted within the context of the Special Operation Ronda Maria da Penha (OERMP), a service that is part of the Network for Combating Violence Against Women, aimed at monitoring women in situations of domestic and family violence who have an Emergency Protective Measure.

### Data source

In accordance with the principles of Grounded Theory (GT), in its updated Straussian strand, it is the researchers’ responsibility to select the participants who will compose the first sample group, with the purpose of guiding the theoretical model being constructed^(9,10)^. Considering the object of the study, this group was composed of 16 women. The following inclusion criteria were defined: age 18 years or older and being monitored by the OERMP due to conjugal violence. Exclusion criteria included women who, during data collection, showed emotional instability and those who failed to attend the interview twice consecutively without prior notice. It is noteworthy that there were no cases of withdrawal throughout the study. Initial contact with participants in the first group was facilitated by the Women’s Reflective Group (GRM), a space dedicated to reflection and empowerment for a life free from conjugal violence.

After analyzing the data collected from the first group, the study hypothesis was outlined, which guided the formation of the second sample group, composed of ten health professionals - three physicians, five nurses, and two psychologists - working in the Family Health Strategy of a Health District in Salvador, Bahia, Brazil. Professionals were selected based on the following inclusion criterion: having worked in the FHS for at least six months. This time requirement was established to ensure a minimum level of connection between the professional and the community. Exclusion criteria included professionals on vacation, leave, or medical absence; only one professional was excluded due to maternity leave. Theoretical saturation was reached within the second sample group, making further data collection unnecessary to support the emerging phenomenon.

The strategy for approaching this second group involved activities directed at health professionals working in the FHS of the Sanitary Suburb Health District (SSHD), in Parque São Bartolomeu. These activities addressed themes related to violence, fostering integration with the professionals and contributing to data collection from this second sample group.

### Data collection and organization

Data were collected through individual interviews lasting an average of one hour and thirty minutes, conducted between December 2020 and July 2021 via the Google Meet video calling platform. It was not necessary to conduct more than one interview with each participant. The interviews were conducted by doctoral students and graduate fellows with experience in qualitative research. A semi structured form was used, containing objective questions related to the participants’ sociodemographic characteristics and the guiding question: “How do women with a history of intimate partner violence experience sexuality?”. Based on participants’ responses, additional questions emerged throughout the interviews, such as: “Do you think your experience of sexuality and sex has impacted your life and health? In what way?” among others. These follow up questions aimed to keep the interviews focused on the study object and to achieve depth in the data regarding the dimensions and progression of the research.

All interviews were audio recorded and later transcribed verbatim. The material was organized according to the three coding stages - open, axial, and integration - with support from NVivo 10 software. The criterion of data saturation was adopted to determine the end of data collection.

### Data analysis

Thus, codes were established that gave rise to categories and guided the development of the hypothesis: *“Women with a history of intimate partner violence are socialized to experience a non pleasurable and repressed sexuality, which compromises their physical and mental integrity and leads them to seek professionals working in the FHS”.*

The axial coding process was guided by the analytical tool known as the *Paradigmatic Model*, through which analytical categories were organized into three components: Conditions, Actions-Interactions, and Consequences. From the integration of these elements, the phenomenon corresponding to the study’s central category emerged: *“Constructing female sexuality through assumptions of a sexist culture with impacts on life and health”.* This central category was validated by participants from one of the sample groups and by experts in Grounded Theory. It is important to note that, in the present article, the researchers focused on presenting the categories related specifically to the consequences of the phenomenon under study.

## RESULTS

The 17 women who participated in the study were between 29 and 61 years old, with a mean age of 46 years; 94% (n=16) self identified as Black, 94% (n=16) reported having a religion, 58.8% (n=10) had completed high school, and 94% (n=16) were engaged in paid work. Regarding the duration of the abusive relationship, 41% (n=7) experienced violence from their former partner for 11 to 20 years, and 70.5% (n=12) had up to two children.

Among the ten health professionals included in the study, seven identified as female and three as male; 80% (n=8) held postgraduate degrees in Family Health and/or related fields, and 60% (n=6) had been working in the Family Health Strategy for more than six years.

### Experiencing repercussions on physical health

#### Feeling pain during sexual intercourse

The painful sensation during sexual intercourse was present in the reports of women with a history of intimate partner violence and was even associated with episodes of vaginal bleeding. This experience of sexuality is linked to difficulty achieving pleasure and/or orgasm, related to muscle tension and lack of lubrication of the vulva and vagina.

*It was very painful because I didn’t feel any pleasure, and sometimes I even bled. My muscles weren’t relaxed and I wasn’t lubricated.* (M9)

*Any inhibition of sexuality results in difficulty experiencing pleasure and reaching orgasm, which contributes to the sensation of pain during sexual intercourse.* (ME3)

#### Experiencing the impacts of unsafe sex

The data indicate that, throughout their lives, women experience a sexuality marked by a lack of knowledge about contraceptive methods and the prevention of Sexually Transmitted Infections (STIs), resulting in repeated unplanned and/or unwanted pregnancies and in illness caused by STIs. These consequences are further aggravated by male power exerted over women’s bodies, including the partner’s refusal to undergo STI treatment and his imposition of abortion.

*I got pregnant the first time I had sex because I didn’t know how to prevent it.* […] *He gave me a medication to induce an abortion; I lost a lot of blood and almost died. To this day, I feel guilty. After that, I had six more daughters, all unplanned.* […] *He also transmitted a bacterial infection to me through sex and refused to get treated, saying that I had passed it to him.* (M3)

*They usually get pregnant during their first sexual experiences.* […] *One of them underwent an abortion and ended up developing an infection. They frequently present with STIs, such as syphilis, including recurrent cases. We try to bring the partners in for treatment, but it’s difficult.* (E3)

### Experiencing repercussions on physical health

#### Presenting physic suffering

Over the course of their lives, women reveal that experiencing past sexual violence and/or violence within intimate relationships has resulted in psychological suffering, expressed, for example, through depressed mood and loss of motivation to live. In this context, the study highlights the need for specialized professionals to address the demands related to mental health.

*The experiences of sexual abuse I suffered during adolescence and within my marriage left me very sad and depressed. I no longer recognized myself as before, and because of that I kept trying to find myself again. It took me a long time to find meaning in my life. I felt better after starting therapy with a psychologist.* (M15)

*Experiencing violence in sexual relations also leads to psychological impairment. I notice that the women arrive with their heads down and avoid looking me in the eyes during the consultation. They are saddened, depressed, and may even require treatment.* (P2)

#### Feeling uncomfortable with their own body

The statements point to an experience of female sexuality weakened by the imposition of aesthetic standards on their bodies, rooted in the social imagination and reinforced by their partners. These stereotypes generate embarrassment and shame in women, associated with low self-esteem, contributing to excessive self criticism and the development of strategies to hide themselves during sexual activity.

*I don’t consider myself a woman who feels comfortable enough with my body to believe that having the lights on wouldn’t interfere with sexual intercourse. I don’t have the self confidence to let my partner look at me. I find ways to avoid it: turning off the lights, covering myself. He also criticized my body, which made my self esteem even worse. I had to go to therapy to feel more secure.* (M2)

*In the clinic, some of them say their intimate parts are ugly; they worry about their appearance. They don’t show their bodies in the light and only have sex in the dark. Even for routine gynecological exams, they feel ashamed and embarrassed.* (E5)

### Experiencing repercussions on the relatioship

#### Feeling repulsion after giving in to sex

The participants describe engaging in sexual activity without desire in response to their partner’s insistence. Giving in to such situations generates feelings of guilt and disgust, which reverberate into repulsion toward their own bodies, to the point that they struggle to face their own reflection in the mirror, often resorting to showering as a way to cleanse themselves of these sensations.

*He insisted a lot, and I ended up having sex even without wanting to. Even though I felt a lot of guilt for giving in, it was better than dealing with his insistence. When it was over, I would go straight to the shower. I felt disgusted even looking at myself in the mirror, at facing myself. I felt repulsion.* (M16)

*She had sexual relations with her husband even when she didn’t want to, because she felt relieved to get rid of the pressure. She also reported feeling disgust and rejection, in addition to guilt for putting herself in that situation.* (E2)

#### Creating strategies to avoid sexual activity

Sexual intercourse devoid of pleasure, which is recurrent in the sexual experiences of women with a history of intimate partner violence, results in a reduction of libido. This decrease in sexual desire leads women to develop different strategies to avoid the act.

*I completely lost sexual interest in him. My libido just kept decreasing. Sometimes I pretended I was on my period so I wouldn’t have intercourse, or that I needed to urinate at the moment of sex.* (M4)

*The fact that it isn’t pleasurable leads to a decrease in libido, desire, and willingness to have intercourse with the partner. They end up using excuses such as menstruation, headaches, body pain, tiredness, and sleepiness to avoid sex.* (ME1)

#### Blocking oneself during sexual relations

In their accounts of their sexual experiences, the women indicate that, although they wish to relate to other people after ending an abusive relationship, they report difficulties when it comes to expressing sexual interest. This is connected to previous negative experiences, which generate distress and emotional blockage in their current relationships.

*I feel the need to have a partner, but this is still complicated for me. I can go on a date, but when I notice any sexual interest, I pull away. I can’t give myself or allow myself to have a new sexual experience, and that makes me anxious. I feel blocked* [cries].(M5)

*They lose their self-esteem and, because they associate sex with something negative, they shut down. I’ve had a patient who said she never wanted to have sex with anyone again.* (E3)

### Experiencing the discovery of sexual pleasure

#### Discovering pleasure with one’s own body

In light of the distressing sexual experiences within a violent marital context, the women turn to bodily self knowledge strategies to achieve sexual satisfaction. Their accounts highlight the importance of health professionals engaging in dialogue about recognizing erogenous zones as a pathway to pleasure, including the possibility of using erotic products such as vibrators and practicing masturbation.

*Since I didn’t feel pleasure with him, the doctor advised me to start stimulating myself.* […] *I began by buying devices at a sex shop. I felt good with my little toys* [vibrators] *and with my own intimacy; I started to recognize the areas of greater sensitivity and arousal.* (M12)

*Masturbation has a positive impact on women’s quality of life. It allows them to identify the areas where they feel the most pleasure, and this brings improvements to their sexual relationships.* (E4)

#### (Re) Discovering pleasure in sexual intercourse

Some women have the opportunity to experience new and healthy intimate relationships despite their past histories of intimate partner violence. What stands out is the progress they make in communicating with their partners, guiding them in how to touch in ways that enhance their pleasure, which even facilitates reaching orgasm.

*I talked to my current partner and told him that I was coming from a violent relationship and that having sex was a problem for me. He said we could do things at my pace and in the way I felt comfortable.* […] *I would always tell him how I liked to be touched, and I began to have orgasms.* (M1)

*Women who start talking to their partners about their sexual preferences find it easier to reach pleasure.* (ME1)

## DISCUSSION

The sexual experiences of women with a history of intimate partner violence have repercussions on their health, such as pain during sexual intercourse. Dyspareunia, which is sometimes accompanied by vaginal bleeding as evidenced in the study, is often linked to sexual practices that generally occur in the absence of desire and in the presence of force^(14)^. The hurried manner in which sexual intercourse is performed hinders the unfolding of the female sexual response cycle, since phases such as desire, arousal, muscle relaxation, and lubrication are disregarded by the partner^(15,16)^. As a result, these women continue to experience sexual relations that distance them from pleasure and orgasm.

In this context, the decrease or absence of sexual desire is a concern frequently reported by women during consultations with healthcare professionals. However, in an attempt to address the complaint, professionals sometimes act in a limited way, recommending the use of vaginal lubricants and/or hormonal treatments^(4,17)^. A more effective approach would involve a careful investigation of the underlying cause that triggered the complaint, as this would allow for the diagnosis of intimate partner violence, sexual dysfunctions, or other issues related to sexuality^(18,19)^.

It is important to understand that forced sexual intercourse within the context of marriage, classified as marital rape and therefore devoid of pleasure, results in decreased sexual interest and reduced libido. Rape culture has been reinforced by the patriarchal social institution over the centuries, consolidating a perspective of male dominance that manifests in the belief that men may assert their superiority through coercion and physical force. The violence experienced by wives emerges from the notion that they cannot refuse such acts, as their bodies are conditioned to serve as objects for their spouses^(3)^.

It is noteworthy that situations of sexual violence - whether experienced in adulthood within intimate relationships or occurring during childhood and/or adolescence - impact women’s sexual lives, a reality reflected in the findings. Desires, behaviors, sexual activity, and intimate contact are marked by the possibility of recalling past traumas, which may act as triggers for psychological distress. In this regard, a study conducted in the southern United States with 1,213 women showed that participants with experiences of childhood sexual abuse reported lower sexual satisfaction compared to women without a history of domestic violence^(20)^. Therefore, it is essential that healthcare professionals conduct a detailed anamnesis that includes aspects of sexuality and facilitates the identification of nuances related to traumatic experiences and dysfunctional relationships^(19-21)^.

Immersed in this reality, women who have been victims of unwanted and coerced sex may develop a repertoire of strategies to avoid the act and protect themselves from the resulting suffering, using pretexts such as headache, menstruation, or sleep. These strategies, mentioned by the participants, must be employed due to the difficulty women face in saying no, since sex is perceived by both partners as a marital obligation^(2)^. The normalization of sex as a woman’s responsibility - although it constitutes an oppressive experience within their relationships - is identified in studies as an element that women themselves do not always label as violence. In this sense, it becomes evident that the patriarchal and sexist context continues to hinder women’s ability to verbalize their experience of this harm^(22)^.

Faced with the demands of marital commitment, the women in the study give in to their partner’s desire, caresses, and expected roles, even when they feel guilty for doing so. After the occurrence, they struggle to look at themselves, as they feel dirty and begin to adopt behaviors aimed at bodily purification, such as taking a shower^(22,23)^. Similarly, this feeling - marked by repulsionalso occurs in situations of rape perpetrated by strangers^(24)^.

The dominance of men over women’s desires extends to other aspects of sexual and reproductive life, such that women have their individual choices managed by their partners^(8,25,26)^. In this sense, male power manifests itself through restrictions on the use of contraceptive methods, refusal to use condoms for the prevention of STIs, and the imposition that the partner undergo an abortion in the case of an unwanted pregnancy. These situations reflect mechanisms of control over women’s bodies - understood as objects - within the institution of marriage, which legitimizes men’s exclusive sexual possession of women in order to meet their own needs^(3)^.

Male supremacy also authorizes men to pass judgment on women’s bodies. According to the women interviewed, they are ridiculed and have their flaws pointed out, which affects their self-esteem, intensifies body-related discomfort, and contributes to feelings of inadequacy during sex. This dynamic persists due to a social construct of stereotyped, unrealistic bodies - often far removed from what is natural - yet considered ideal and desired by both men and women^(3,27)^. The commercial appeal of achieving the “perfect body” is so strong that an entire industry exists to reshape bodies, offering surgical procedures in various areas, including the remodeling of women’s genital anatomy, which may compromise their sexual functioning^(28)^.

The feeling of bodily inadequacy, linked to the experience of violent sexual practices, leads women to block themselves from pleasure, libido, emotions, and sex itself. This reality is also observed when these women enter new relationships, identified in the findings as an impact on their sexuality, similar to what has been reported in studies conducted in Paraná^(29)^. In this context, some women attempt to seek new partners but, when faced with the possibility of deeper involvement, intimacy, and sexual activity, they withdraw out of fear of reliving past experiences of violence, disrespect, imposition of unwanted practices, among others.

Faced with these difficulties in establishing relationships, women may resort to sexual satisfaction practices that do not depend on another person, such as masturbation. Self-eroticism is a strategy to stimulate pleasure, promote relaxation, and generate a sense of well-being^(30-32)^. This practice supports bodily self-knowledge of erogenous zones and may be associated with the use of erotic products^(30)^. While some women benefit from these resources, others may not feel comfortable or may not experience pleasure in using them, making free choice essential^(31)^.

Regarding the benefits of masturbation, one finds an improvement in women’s self-image^(30)^. Through the manipulation of their bodies and genitals, they can recognize the potential of a body that was once rejected and can reclaim the capacity it holds. In this dynamic, women may become more empowered in relation to their sexuality, even managing to speak more spontaneously about their desires and expectations within relationships^(30,32,33)^.

In this sense, it is important that professionals - especially those in nursing - remain attentive to guiding women about possibilities for self-knowledge and self-pleasure, contributing to improvements in their quality of life, since the practice goes beyond touch and is related to intimate discoveries that help them feel well with themselves, independently of another person. This process of dialogue with women, which can be facilitated in Primary Health Care (PHC) settings, aims at the normalization of sexuality and represents a challenge to be incorporated into healthcare encounters, given the limiting beliefs that still prevail in society.

### Study limitations

The study is limited by not having investigated, as a consequence, the possibility of behavioral changes related to the taboos learned throughout life by women with a history of intimate partner violence. In this sense, it was not possible to determine whether they sought to break these patterns in the upbringing, for example, of their daughters or sons.

### Contributions to the area of nursing

The study offers elements to support nursing professionals in the investigative process regarding the repercussions on women’s lives and health related to the exercise of their sexuality, especially during consultations in which the topic is more likely to be addressed, such as reproductive planning, cervical and breast cancer screening, or even during prenatal care. This focus within consultations requires a welcoming, non judgmental professional approach, guiding women toward new horizons of self knowledge and autonomy in the exercise of their sexuality. In addition to these settings, nursing can make use of other interaction spaces within Primary Health Care (PHC) that reinforce women’s autonomy in the realm of sexuality, such as community discussion groups, public schools, health fairs, and educational activities in waiting rooms.

## FINAL CONSIDERATIONS

The study showed that the way women with a history of intimate partner violence experience their sexuality results in repercussions such as dyspareunia, sexually transmitted infections, unplanned pregnancies, psychological distress, and discomfort with their own bodies the latter stemming from the depreciation imposed by their partners. By giving in to sex, women develop feelings of repulsion and create strategies to avoid it, a dynamic that has triggered barriers to forming new relationships.

These experiences motivate women to seek self knowledge and self pleasure, using bodily touch, including masturbation. Guidance from health professionals in demystifying taboos surrounding this possibility is highly valuable, as it can contribute to the process of (re)discovering pleasure with oneself and with others. In this sense, attention is drawn to the engagement of these professionals in investigating the repercussions women present in their sexuality, their causes, and the possibilities for progress toward restoring a healthy sexual life.

Furthermore, considering the specificities involved in reaching women with a history of intimate partner violence, it becomes important to create spaces for interaction between them and the health service, such as reflective groups, with the purpose of demystifying taboos related to female subservience in relationships an expectation that has historically directed women to focus their efforts solely on ensuring male pleasure. Thus, creating spaces for dialogue that support women’s empowerment regarding the freedom to exercise their sexuality centered on pleasure and sexual self satisfaction is essential in the process of transforming social and sexual behaviors. These practices may help prevent negative repercussions related to the exercise of sexuality and promote quality of life and health for women.

## Data Availability

The research data are available within the article.
