# Supplementary material for: Repercussions of the sexual experiences of women with a history of domestic violence: A Grounded Theory
Source: Rev Bras Enferm. 2026 Oct 19;79:e20240561. doi: 10.1590/0034-7167-2024-0561 (PMC13679594; doi:10.1590/0034-7167-2024-0561)
Supplement: Translation

# Translation: Repercussões das experiências de sexualidade de mulheres com história de violência conjugal: Uma *Grounded Theory*

**DOI:** 10.1590/0034-7167-2024-0561pt

**Authors:** Luana Moura Campos, Nadirlene Pereira Gomes, Lilian Conceição Guimarães de Almeida, Jordana Brock Carneiro, Josinete Gonçalves dos Santos, Janli Kelly Pereira Fontes dos Santos, Joyce Mendes Paim, Rebeca dos Santos Santos

**Affiliations:** I Universidade Estadual do Sudoeste da Bahia. Jequié, Bahia, Brasil; II Universidade Federal da Bahia. Salvador, Bahia, Brasil; III Universidade do Estado do Rio de Janeiro. Rio de Janeiro, Rio de Janeiro, Brasil; IV Hospital Sofia Feldman. Belo Horizonte, Minas Gerais, Brasil

**Keywords:** Sexualidade, Violência por Parceiro Íntimo, Análise de Gênero, Saúde da Mulher, Teoria Fundamentada.

## Abstract

**Objetivos:**

conhecer as repercussões das experiências da sexualidade na vida e saúde de mulheres com história de violência conjugal.

**Métodos:**

pesquisa qualitativa, ancorada na *Grounded Theory* e no referencial teórico de gênero e patriarcado conduzido por Kate Millet. Foram entrevistadas 17 mulheres assistidas pela Operação Ronda Maria da Penha, e dez profissionais de saúde da Estratégia de Saúde da Família, em Salvador-BA. Este estudo reúne dados do componente. Consequências do Modelo Paradigmático.

**Resultados:**

foram encontradas as categorias: Experienciando repercussões na saúde física, Experienciando repercussões na saúde psicológica, Experienciando repercussões na relação e Experienciando a descoberta do prazer sexual.

**Considerações Finais:**

as experiências de sexualidade perpassaram por repercussões físicas e psicológicas, como dor no ato sexual, infecção sexualmente transmissível e sofrimento psíquico, além da sensação de desconforto, sentimento de repulsa e bloqueio para as relações sexuais. Apesar disso, algumas mulheres experienciam a descoberta do prazer na relação sexual.

## INTRODUÇÃO

A sexualidade relaciona-se à forma de o sujeito viver no mundo, às interações com o outro, aos desejos e à satisfação sexual^(1)^. Tal experiência sofre influência de diversos aspectos, como a educação, a cultura e os valores patriarcais, podendo impactar na saúde de homens e mulheres de maneira diferente em virtude das desigualdades de gênero^(2)^.

O constructo social de gênero interfere na maneira previsível em que meninos e meninas se relacionam com o mundo, a afetividade, as emoções, o prazer, e por conseguinte a sexualidade. Amparados em uma cultura desigual de gênero, os homens desde a tenra idade são orientados sobre as práticas de satisfação sexual, a priorização dos desejos pessoais e padrões de conduta da masculinidade hegemônica, enquanto para a maioria da população feminina ainda é limitada a oportunidade de aprendizado e experiências que se relacionam à sexualidade, restringindo esse grupo a dar prazer em detrimento de ter prazer^(3-5)^. Essa dicotomia é corroborada por Schweiger^(5)^, em seu estudo sobre socialização de gênero e educação sexual, que destaca a necessidade de uma abordagem de educação sexual sensível ao gênero, que desafie as normas hegemônicas de masculinidade e promova formas alternativas de vivência sexual e afetiva^(5)^.

Para as mulheres, as benesses oriundas de experiências permeadas por fantasias, toques e desejos são roubadas, sendo a relação com o outro reduzida ao propósito da reprodução, ou ainda à satisfação sexual do homem, figura que tem a posse sexual exclusiva da mulher, não havendo reciprocidade^(3,6)^. Logo, aquelas que reconhecem as práticas, os comportamentos e as trocas sexuais como algo que atende às suas necessidades íntimas pessoais, e ignoram as sanções sociais, são julgadas como pecaminosas e vulgares^(7)^. Este comportamento social reprime e coage as mulheres a não explorarem a sua sexualidade, bem como experimentar possibilidades da relação consigo e com o outro^(8)^.

É a partir dessas crenças arraigadas que vão se conformando os relacionamentos íntimos, nos quais mulheres e homens reproduzem padrões de comportamentos aprendidos. Nessa dinâmica, pode haver relações conjugais em que as repressões e sanções acontecem de maneira exacerbada, permeadas pelo desrespeito e violência. O patriarcado sedimenta estas características masculinas, amparado em condutas radicais, súbitas e violentas^(3)^. Tais relacionamentos geralmente são marcados por restrições e pelo sentimento de pertença masculina sobre os corpos das mulheres^(3)^, tornam-se um terreno fértil para experiências negativas no campo da sexualidade^(7,9)^, podendo impactar a vida e saúde.

Assim, diante das inúmeras privações sobre a sexualidade das mulheres, na conjugalidade, prejuízos oriundos de uma sociedade machista e relacionamentos violentos, podem incorrer ao longo da vida situações como depressão, baixa autoestima, isolamento social, entre outros^(10)^. Vale salientar o caráter velado da violência no âmbito da sexualidade, visto que muitas mulheres experienciam as repercussões de maneira silenciosa, o que pode guardar relação com sentimentos como vergonha ou medo do julgamento social, sobretudo quando essa violência é perpetrada dentro de relacionamentos conjugais^(11)^.

Portanto, é essencial atentar para o papel dos profissionais de saúde, especialmente da enfermagem, na identificação das repercussões da violência relacionadas ao campo da sexualidade, a fim de tecer caminhos de apoio às mulheres que vivenciam tal agravo. Desse modo, o ineditismo deste trabalho advém da busca pela compreensão do fenômeno da experiência de sexualidade com mulheres que experimentaram violência conjugal, a fim de despontar para estratégias de lidar com o agravo nos atendimentos de saúde sexual e reprodutiva. Nesse ínterim, observa-se a necessidade de se conhecer sinais que direcionam a atuação do profissional de saúde para a melhoria da qualidade de vida das mulheres no âmbito da sexualidade.

### Relevância do estudo

Este artigo é um produto da Tese Sexualidade de mulheres com história de violência conjugal: compreendendo o fenômeno a partir de uma Teoria Fundamentada nos Dados^(12)^ depositada no repositório institucional https://repositorio.ufba.br/bitstream/ri/38543/1/TESE%20-%20LUANA%20MOURA%20CAMPOS.pdf. Os dados de pesquisa estão disponíveis em repositório.

## OBJETIVOS

Conhecer as repercussões das experiências da sexualidade na vida e saúde de mulheres com história de violência conjugal.

## MÉTODOS

### Aspectos éticos

A pesquisa obteve aprovação do Comitê de Ética em Pesquisa da Escola de Enfermagem da UFBA através do parecer no 2.639.224/2018 e CAAE: 88960217.6.0000.5531 e respeitou as resoluções n^o^ 466/2012 e 510/2016 do Conselho Nacional de Saúde. Com o intuito de preservar a identidade, as mulheres entrevistadas foram identificadas por um código formado pela letra M (refere à palavra “mulher”) seguido do número de ordem da entrevista, exemplo “M1”. No caso dos profissionais de saúde, a codificação associou a letra referente à categoria profissional (enfermeira-E, médica-ME e psicóloga-P) ao algarismo referente à ordem de entrevista. Vale lembrar que o Termo de Consentimento Livre e Esclarecido foi obtido de todas(os) as(os) participantes envolvidas(os) no estudo por meio de documento impresso. Salienta-se que, para assinatura, foram lidas previamente as informações às participantes, que continham os objetivos da pesquisa e o interesse do estudo, bem como o seu processo de condução.

### Referencial teórico-metodológico

Os dados obtidos foram analisados a partir da *Grounded Theory* (GT), mais especificamente a vertente Straussiana atualizada^(13)^, e nos pressupostos teóricos do gênero e patriarcado sob a ótica de Kate Millet, em 1970^(3)^. Tal referencial desenvolveu discussões feministas sobre o gênero sob a perspectiva do que atualmente intitulamos marcadores sociais, englobando as relações com raças, castas, classes e sexos. Chamou ainda atenção a maneira com que aborda o “sexo”, considerando-o enquanto uma categoria que sofre influência política, sendo, portanto, eixo que consolida o sistema patriarcal enquanto instituição de controle^(3)^.

### Tipo de estudo

Trata-se de um estudo de abordagem qualitativa vinculado ao projeto matriz: “Enfrentamento da violência conjugal no âmbito do Sistema Único de Saúde: tecnologia social envolvendo mulheres, homens e profissionais da Atenção Básica”, submetido ao edital Programa de Pesquisa para o SUS: Gestão Compartilhada em Saúde - PPSUS/BA. Para isso, para relatar a pesquisa qualitativa, utilizou-se o guia *Consolidated Criteria for Reporting Qualitative Research* (COREQ). A equipe responsável pela coleta de dados foi composta por seis dos oito autores do estudo, sendo mulheres com diferentes níveis de formação acadêmica, incluindo graduandas, mestrandas e doutoras em Enfermagem. Todas tinham experiência prévia em conduzir entrevistas qualitativas e atuavam como enfermeiras e bolsistas de iniciação científica. Essa vivência prévia proporcionou maior sensibilidade ao abordar questões íntimas e delicadas relacionadas ao tema da pesquisa.

### Pressuposto Teórico

Mulheres com história de violência conjugal recebem uma educação sexual enraizada no machismo estrutural e experienciam a sua sexualidade de forma não prazerosa e reprimida, pautando-a na satisfação do cônjuge, o que compromete a sua integridade física e mental e a faz buscar profissionais que atuam na Estratégia de Saúde da Família (ESF). Essa situação apontou para a necessidade de investigação juntamente a um segundo grupo amostral, composto por estes profissionais.

### Cenário de Estudo

A presente pesquisa foi desenvolvida no cenário da Operação Especial Ronda Maria da Penha (OERMP), serviço que integra a Rede de Enfrentamento à Violência contra as Mulheres, com a finalidade de acompanhar mulheres em situação de violência doméstica e familiar que possuem Medida Protetiva de Urgência.

### Fonte de dados

Atendendo aos pressupostos da GT, em sua vertente Straussiana atualizada, é de responsabilidade das pesquisadoras elencar as participantes que irão compor o primeiro grupo amostral, com a finalidade de conduzir o modelo teórico que está sendo construído^(9,10)^. Considerando o objeto de estudo, tal grupo foi composto por 16 mulheres. Foram definidos os seguintes critérios de inclusão: idade igual ou superior a 18 anos e acompanhamento pela OERMP em virtude da violência conjugal. Foram adotados como critérios de exclusão aquelas mulheres que, durante a coleta de dados, se mostraram emocionalmente instáveis e as que não compareceram ao momento da entrevista sem comunicação prévia por duas vezes consecutivas. Salienta-se que não houve casos de desistência ao longo da pesquisa. A aproximação inicial com as participantes do primeiro grupo foi facilitada pelo Grupo Reflexivo de Mulheres (GRM), um espaço dedicado à reflexão e empoderamento para uma vida livre de violência conjugal.

Após a análise dos dados coletados no primeiro grupo, foi delineada a hipótese do estudo, a qual conduziu para o segundo grupo amostral, sendo composto por dez profissionais de saúde - três médicas(os), cinco enfermeiras(os) e duas psicólogas que atuam na Estratégia de Saúde da Família de um Distrito Sanitário de Salvador, Bahia, Brasil. Os profissionais foram selecionados com base no seguinte critério de inclusão: estar atuando no âmbito da ESF a um período igual ou superior a seis meses. A medida temporal foi estipulada com vistas a assegurar que o profissional de saúde estivesse com o mínimo de vínculo com a comunidade. Para exclusão, adotou-se profissionais que estivessem afastados por motivo de férias, licença ou questões de saúde, sendo que uma profissional apenas não integrou o estudo em virtude de licença maternidade. Alcançou-se a saturação teórica dos dados no segundo grupo amostral, não sendo necessária a investigação de novas informações para dar sustentação ao fenômeno encontrado.

A estratégia de aproximação desse segundo grupo foi a realização de ações voltadas aos profissionais de saúde vinculados à ESF do Distrito Sanitário Subúrbio Ferroviário (DSSF), no parque São Bartolomeu. Essas atividades abordavam temáticas relacionadas à violência, promovendo integração com os profissionais e contribuindo para a coleta de dados a partir de um segundo grupo amostral formado por profissionais de saúde.

### Coleta e organização dos dados

Os dados foram coletados por meio de entrevistas individuais com duração média de uma hora e trinta minutos realizadas entre os meses de dezembro de 2020 a julho de 2021, por meio da plataforma de videochamadas *Google Meet*. Não foi necessário realizar mais de uma entrevista com as participantes. Esse momento foi conduzido por doutorandas, bolsistas do Programa de Pós-Graduação, com experiência na abordagem qualitativa, sendo utilizado formulário semiestruturado contendo questões objetivas referentes à caracterização sociodemográfica das participantes e à pergunta norteadora: “Como as mulheres com história de violência conjugal experienciam a sexualidade?”. Com base nas respostas das participantes, outras possibilidades de perguntas foram surgindo ao longo das entrevistas, como por exemplo: “Você acha que a sua vivência de sexualidade e sexo impactaram na sua vida e saúde? De que forma?”, dentre outras. Essas derivações possuem o intuito de tornar as entrevistas mais focadas no objeto estudado e de se alcançar profundidade dos dados no que concerne às dimensões e avanço da pesquisa.

As falas foram gravadas e posteriormente transcritas na íntegra, sendo organizadas atendendo às três etapas de codificação - aberta, axial e integração e com o suporte do software NVIVO10. Foi adotado o critério de saturação de dados para encerramento da coleta de dados.

### Análise dos dados

Assim, foram estabelecidos códigos que deram origem a categorias e direcionaram para a hipótese: “mulheres com história de violência conjugal são educadas para experienciar uma sexualidade não prazerosa e reprimida, o que compromete a sua integridade física e mental e as leva a buscar profissionais que atuam na ESF”.

O processo de codificação axial foi norteado pela ferramenta de análise intitulada Modelo Paradigmático, a partir da qual foram ordenados em categorias analíticas atreladas a três componentes: Condições, Ações-interações e Consequências. Da integração destes elementos emergiu o fenômeno que corresponde à categoria central do estudo: “Construindo a sexualidade feminina por meio de pressupostos da cultura machista com impactos para a vida e saúde”, validado por participantes que integraram um dos grupos amostrais e experts na GT. Destaca-se que no presente trabalho as pesquisadoras ativeram-se a apresentar as categorias que dizem respeito às consequências do fenômeno estudado.

## RESULTADOS

As 17 mulheres que fizeram parte do estudo têm idade entre 29 e 61 anos, sendo a média correspondente a 46 anos; 94% (n=16) se autodeclaram da raça negra, 94% (n=16) têm religião, 58,8% (n=10) concluíram o ensino médio e 94% (n=16) dessas trabalham de forma remunerada. No que tange ao tempo de permanência na relação abusiva, 41% (n=7) experienciaram de 11 a 20 anos a violência praticada pelo seu ex-companheiro, e 70,5% (n=12) tinham até dois filhos.

Dos dez profissionais de saúde investigados, sete se declararam do sexo feminino e três do sexo masculino, 80% (n=8) possuem pós-graduação nas áreas de Saúde da Família e/ou áreas afins, e no que tange ao tempo de atuação na ESF 60% (n=6), trabalhavam há mais de seis anos.

### Experienciando repercussões na saúde física

#### Sentindo dor no ato sexual

A sensação dolorosa durante o ato sexual esteve presente no relato das mulheres com história de violência conjugal, associada, inclusive, a episódios de sangramento vaginal. Essa experiência da sexualidade atrela-se à dificuldade de obtenção de prazer e/ou orgasmo relacionado ao tensionamento da musculatura e ausência de lubrificação da vulva e vagina.

*Era muito doloroso porque não tinha prazer, algumas vezes até sangrava. Meus músculos não estavam relaxados e eu não ficava lubrificada.* (M9)

*Qualquer inibição de sexualidade reflete na dificuldade de ter prazer e de gozar, o que colabora com a sensação de dor no ato sexual.* (ME3)

#### Vivenciando impactos do sexo inseguro

Os dados apontam que, ao longo da vida, as mulheres vivenciam uma sexualidade que perpassa pelo desconhecimento sobre os métodos contraceptivos e de prevenção de Infecção Sexualmente Transmissível (IST), repercutindo em consecutivas gestações não planejadas e/ou indesejada e no adoecimento por IST. Essas repercussões são agravadas ainda por atitudes de poder masculino sobre o corpo da mulher ante a negativa do cônjuge ao tratamento da IST e a sua imposição à realização do aborto.

*Engravidei na primeira relação, porque não sabia como evitar.* [...] *ele me deu um remédio para abortar, perdi muito sangue e quase morri! Até hoje me sinto culpada. Depois disso, tive mais seis filhas, sem planejamento.* [...] *ele já me passou uma bactéria pelo sexo e se recusou a tratar dizendo que eu tinha passado para ele.* (M3)

*Elas engravidam normalmente nas primeiras relações sexuais.* [...] *uma delas foi submetida ao aborto e acabou tendo infecção. Frequentemente aparecem com IST’s, como sífilis, inclusive de repetição. A gente tenta captar eles* [o parceiro]*, mas é difícil.* (E3)

### Experienciando repercussões na saúde psicológica

#### Apresentando sofrimento psíquico

No decurso da vida, as mulheres revelam que experienciar violência sexual pregressa e/ou a conjugalidade acarretou sofrimento psíquico, expresso, por exemplo, através do humor deprimido e da perda da motivação para viver. Nesse ínterim, o estudo expõe a necessidade de profissionais especializados para lidar com as demandas no campo da saúde mental.

*As vivências de abusos sexuais que sofri na adolescência e dentro do casamento me deixaram muito triste e deprimida. Não me reconhecia como antes e por isso ficava tentando me reencontrar. Demorei para encontrar um sentido para a minha vida. Me senti melhor depois de começar terapia com uma psicóloga.* (M15)

*Vivenciar violência no sexo também traz comprometimento psicológico. Percebo que as mulheres chegam cabisbaixas, não olham nos meus olhos durante o atendimento. Elas estão entristecidas, deprimidas, e podem até demandar tratamento.* (P2)

#### Sentindo-se desconfortável com o próprio corpo

As falas direcionam para uma experiência da sexualidade feminina fragilizada diante da imposição de padrões estéticos sobre o seu corpo contidos no imaginário social e reforçados pelos cônjuges. Estes estereótipos geram nas mulheres constrangimento e vergonha associados à baixa autoestima, contribuindo para a autocobrança excessiva e elaboração de estratégias para se esconder durante o sexo.

*Não me considero uma mulher bem resolvida com o meu corpo a ponto de achar que a luz acesa não vai atrapalhar a relação sexual. Não tenho essa autoconfiança do parceiro ficar me olhando. Dou um jeito de burlar: apagar a luz, me cobrir. Ele também criticava meu corpo, o que piorava minha autoestima. Tive que fazer terapia para me sentir mais segura*. (M2)

*No consultório algumas dizem que as partes íntimas são feias, ficam preocupadas com a estética. Não mostram seus corpos no claro, só transam no escuro. Até para fazer o preventivo se sentem envergonhadas, constrangidas.* (E5)

### Experienciando repercussões na relação

#### Sentindo repulsa após ceder ao sexo

As participantes mencionam a realização do sexo sem vontade frente à insistência do cônjuge. Ceder a tal situação gera nas mulheres, sentimentos de culpa e nojo reverberando na repulsa pelo próprio corpo, a ponto de terem dificuldade de encarar a própria imagem refletida no espelho, levando-as ao banho para depurar essas sensações.

*Ele insistia muito e eu acabava fazendo sexo mesmo sem vontade. Mesmo sentindo muita culpa por ceder, era melhor do que lidar com a insistência dele! Quando acabava ia logo para o banho. Ficava com nojo até de me olhar no espelho, de me encarar. Sentia repugnância.* (M16)

*Ela tinha relações com o marido mesmo sem querer, pois ficava aliviada por se livrar da cobrança. Relatou também que sentia nojo e repúdio, além da culpa por se colocar nesta situação.* (E2)

#### Criando subterfúgios para evitar o sexo

A relação sexual desprovida de prazer, recorrente na experiência da sexualidade de mulheres com história de violência conjugal, culmina na redução da libido. Esta diminuição do desejo sexual leva as mulheres a elaborarem diferentes estratégias para evitar o ato.

*Eu perdi totalmente o interesse sexual por ele. A minha libido só diminuía. Às vezes eu fingia que estava menstruada para não ter relações ou que estava com vontade de urinar na hora H.* (M4)

*O fato de não ser bom faz com que diminua a libido, o desejo, a vontade de ter relação com o cônjuge. Acabam usando desculpas como menstruação, dor de cabeça, dor no corpo, cansaço e sono para evitar o sexo.* (ME1)

#### Bloqueando-se nas relações sexuais

Em seus relatos acerca das experiências no âmbito da sexualidade, as mulheres apontam que, apesar de desejarem relacionar-se com outras pessoas após o rompimento com a relação abusiva, referem dificuldades diante da demonstração de interesse sexual. Isso guarda relação com as experiências negativas anteriores, que geram angústia e bloqueio nas relações atuais.

*Sinto necessidade de ter um companheiro, mas isso para mim ainda é complicado. Consigo ir a um encontro, porém quando percebo o interesse por sexo me afasto. Eu não consigo me doar e me permitir ter uma nova experiência sexual e isso me deixa angustiada. Sinto um bloqueio* [choro]. (M5)

*Elas perdem a autoestima e, por atrelarem o sexo a uma coisa negativa, se bloqueiam. Já tive paciente que disse que não queria mais fazer sexo na vida com ninguém.* (E3)

### Experienciando a descoberta do prazer sexual

#### Descobrindo o prazer com o próprio corpo

Diante dos dissabores das experiências da sexualidade no contexto conjugal violento, as mulheres utilizam estratégias de autoconhecimento corporal para conquistar a satisfação sexual. As falas evidenciam a importância de profissionais de saúde dialogarem sobre o reconhecimento de áreas erógenas para obtenção do prazer, incluindo a possibilidade de uso de produtos eróticos como vibradores e da prática da masturbação.

*Como eu não sentia prazer com ele, a médica orientou que eu começasse a me estimular.* [...] *iniciei comprando aparelhos no sex shop. Me sentia bem com meus brinquedinhos* [vibradores] *e minha intimidade, passei a reconhecer as áreas de maior sensibilidade e tesão.* (M12)

*A masturbação tem impacto positivo na qualidade de vida da mulher. Possibilita que ela reconheça os locais onde sente mais prazer, isso traz melhorias para suas relações sexuais.* (E4)

#### (Re) Descobrindo o prazer na relação sexual

Algumas mulheres têm a oportunidade de viver novos relacionamentos íntimos saudáveis, apesar das histórias pregressas de violência conjugal. Chama atenção para o avanço no diálogo delas com o parceiro, a fim de direcionar o toque em busca do seu prazer, inclusive facilitando alcançar o orgasmo.

*Conversei com meu parceiro atual que eu vinha de um relacionamento violento e que transar para mim era um problema. Ele falou que poderíamos fazer no meu tempo e da forma que eu gostasse.* [...] *sempre falava como eu gostava de ser tocada e passei a ter orgasmos.* (M1)

*As mulheres que passam a falar com os parceiros sobre suas preferências no sexo possuem mais facilidade de atingir o prazer.* (ME1)

## DISCUSSÃO

As experiências da sexualidade de mulheres com história de violência conjugal trazem repercussões sobre sua saúde, a exemplo de dor durante o ato sexual. A dispareunia que, por vezes, é acompanhada por sangramentos vaginais, conforme evidenciado no estudo, costuma estar atrelada às práticas sexuais que ocorrem, geralmente, na ausência de desejo e na presença da força^(14)^. A maneira precipitada como o ato sexual é desempenhado dificulta o desencadear do ciclo de respostas sexuais femininas, visto que fases como desejo, excitação, relaxamentos musculares e lubrificação são desconsideradas pelo cônjuge^(15,16)^. Assim, elas seguem experimentando relações sexuais que as distanciam do prazer e do gozo.

Nesse contexto, a diminuição ou ausência do desejo sexual é uma demanda recorrentemente apontada pelas mulheres durante as consultas com os profissionais de saúde. Contudo, eventualmente, no intuito de sanar a queixa, o profissional atua de maneira pontual, orientando o uso de lubrificantes vaginais e/ou realização de tratamentos hormonais^(4,17)^. Entretanto, uma conduta mais assertiva seria a investigação cuidadosa da causa base que originou a queixa que implica nestas condições, pois assim possibilitaria o diagnóstico das situações de violência conjugal, disfunções sexuais ou outras questões que permeiam a sexualidade^(18,19)^.

É importante compreender que a relação sexual forçada no contexto da conjugalidade, classificada enquanto estupro marital e, portanto, desprovida de prazer, culmina na diminuição do interesse sexual e redução da libido. A cultura do estupro vem sendo sedimentada pela instituição social patriarcal ao longo dos séculos, consolidando a ótica de domínio masculino, que se exprime na concepção de que o homem pode reivindicar sua superioridade através do uso da coerção e força física. A violência sofrida pelas esposas emerge do entendimento de que não poderiam fugir deste ato, já que seus corpos são condicionados a servir como objetos para os cônjuges^(3)^.

Salienta-se que as situações de violência sexual, de maneira geral, sejam aquelas experienciadas quando adultas na conjugalidade ou as que ocorreram ainda na infância e/ou adolescência, e que impactam na vivência da sexualidade das mulheres, realidade apontada nos achados. Os desejos, os comportamentos, o ato sexual e o contato mais íntimo são marcados pela possibilidade de rememorar traumas vividos, podendo ser considerados gatilhos para o sofrimento psíquico de mulheres. Quanto a isso, estudo desenvolvido no Sul dos Estados Unidos da América com 1.213 mulheres demonstrou que as participantes com experiências de abuso sexual infantil relataram menor satisfação sexual em comparação com mulheres sem histórico de violência doméstica^(20)^. Portanto, alerta-se para a importância de profissionais de saúde realizarem uma anamnese detalhada que contemple aspectos da sexualidade e favoreça a identificação de nuances atreladas a vivências traumáticas e relações disfuncionais^(19-21)^.

Imersas nesta realidade, as mulheres que foram vítimas do sexo indesejado e cedido poderão elaborar um repertório de estratégias para esquivar-se do ato e defender-se do sofrimento advindo, usando para isso pretextos como cefaleia, menstruação e o sono. Esses recursos mencionados pelas participantes precisam ser acionados diante da dificuldade das mulheres em dizer não, uma vez que o sexo é tido para ambos enquanto uma obrigação marital^(2)^. A naturalização do sexo enquanto responsabilidade da mulher, embora seja uma vivência opressiva em seus relacionamentos, é apontada por estudo como um elemento não nomeado por elas como violência. Nesse sentido, é evidente que a conjuntura machista-patriarcal permaneça dificultando a verbalização das mulheres sobre a vivência desse agravo^(22)^.

Diante das imposições do compromisso matrimonial, as mulheres do estudo cedem ao desejo, às carícias, e desempenham papéis esperados pelo cônjuge, ainda que se sintam culpadas por isso. Após a ocorrência, as mulheres têm dificuldade de olhar para si, pois se sentem sujas e passam a adotar comportamentos em busca de purificação corporal, usando como recurso, por exemplo, o banho^(22,23)^. De modo semelhante, esta sensação, que agrega repulsa, acontece também em situações de vivência de estupro perpetrado por estranhos^(24)^.

O domínio dos homens sobre os desejos femininos ecoa para outros aspectos da seara sexual e reprodutiva, de maneira que as mulheres têm suas escolhas individuais gerenciadas pelos parceiros^(8,25,26)^. Nesse sentido, o poderio masculino se apresenta desde a restrição para o uso de métodos contraceptivos, a recusa para uso de preservativo com fins na prevenção de IST, bem como a imposição para que a parceira se submeta ao aborto diante da gravidez indesejada. Estas situações refletem os mecanismos de controle sobre o corpo das mulheres, entendidos enquanto objetos, pela instituição casamento, que valida ao homem a posse sexual exclusiva das mulheres com fins a atender às suas necessidades^(3)^.

A supremacia masculina também autoriza os homens a emitirem juízo de valor sobre o corpo feminino. Nesse sentido, eles ridicularizam e apontam defeitos, conforme referido pelas mulheres, o que impacta na autoestima delas, potencializando os desconfortos com o corpo e favorecendo que se sintam desajustadas durante o sexo. Essa situação mantém-se em virtude de um constructo social acerca de corpos estereotipados, irreais, muitas vezes distantes do natural, mas considerados ideais e almejados por homens e mulheres^(3,27)^. O apelo comercial para o alcance do corpo perfeito é tamanho que existe uma indústria voltada para adequar corpos, a qual oferta procedimentos cirúrgicos em diversas áreas, incluindo o remodelamento da anatomia genital da mulher, o que pode comprometer a sua potência sexual^(28)^.

O sentimento de inadequação do corpo, atrelado à experiência da prática sexual violenta, favorece que mulheres se bloqueiem em relação ao prazer, a libido, as emoções e o sexo em si. Essa realidade é observada também quando essas mulheres estabelecem novos relacionamentos, sendo apontado nos achados como um impacto para a sexualidade das mulheres, semelhante ao encontrado em pesquisas desenvolvidas no Paraná^(29)^. Nessa seara, algumas mulheres tentam buscar outras parcerias mas, na iminência de um maior envolvimento, intimidade e sexo, afastam-se por medo de rememorar as vivências do passado como violência, desrespeito, imposições de práticas indesejadas, entre outras.

Frente a essa dificuldade de se relacionar, as mulheres podem recorrer a práticas de satisfação sexual que independem de outra pessoa, como a masturbação. O autoerotismo é uma tática para estimular o prazer, que propicia o relaxamento e promove sensação de bem-estar^(30-32)^. Esta prática favorece o autoconhecimento corporal das zonas erógenas e pode ser associada à utilização de produtos eróticos^(30)^. Embora algumas mulheres se beneficiem do uso destes recursos, outras podem não se sentir confortáveis ou não sentir prazer na sua utilização, sendo essencial a livre escolha^(31)^.

Ainda sobre os benefícios da masturbação, tem-se a melhoria da autoimagem feminina^(30)^. A partir da manipulação do corpo e da genitália elas podem vislumbrar as potencialidades daquele corpo que um dia foi rechaçado e podem usufruir da capacidade que ele tem. Em tal dinâmica, percebe-se que a mulher pode se tornar mais fortalecida sobre a sua sexualidade, conseguindo, inclusive, falar de forma mais espontânea sobre seus desejos e anseios nas relações^(30,32,33)^.

Nesse sentido, é importante que os profissionais, sobretudo da enfermagem, estejam atentos para orientar as mulheres sobre as possibilidades de autoconhecimento e auto prazer, contribuindo para melhoria da sua qualidade de vida, visto que a prática vai além do toque e está relacionada às descobertas íntimas para se sentirem bem consigo, independente do outro. Este processo de diálogo com as mulheres, que pode ser favorecido em ambientes da Atenção Primária à Saúde (APS), tem por finalidade a naturalização da sexualidade, sendo, portanto, um desafio a ser instituído nos atendimentos no cenário da saúde, frente às crenças limitantes ainda preponderantes no seio social.

### Limitação do estudo

O estudo limita-se por não ter investigado enquanto consequência a possibilidade de mudança de comportamentos direcionados pelos tabus apreendidos, ao longo da vida, pelas mulheres com história de violência conjugal. Nesse sentido, não foi possível captar se buscaram a quebra desses padrões na educação, por exemplo, das suas filhas(os).

### Contribuições para a área da Enfermagem

O estudo oferece elementos para subsidiar a prática dos profissionais de enfermagem no processo de investigação sobre as repercussões na vida e saúde das mulheres relacionadas ao exercício da sua sexualidade, sobretudo durante as consultas com maior possibilidade de se tratar sobre o assunto, como o planejamento reprodutivo, de rastreamento do câncer de colo do útero e mamas, ou até mesmo durante o pré-natal. Este direcionamento nas consultas demanda uma abordagem profissional acolhedora e livre de tabus, a fim de conduzir as mulheres a outros horizontes no que tange ao autoconhecimento e autonomia no exercício da sua sexualidade. Além desses ambientes, a enfermagem pode lançar mão de outros espaços de interação na APS que reforçam a autonomia das mulheres no âmbito da sexualidade, como rodas de conversa comunitária, escolas públicas, feiras de saúde e atividades educativas em salas de espera.

## CONSIDERAÇÕES FINAIS

A pesquisa apontou que a forma como mulheres com história de violência conjugal experienciam sua sexualidade lhes traz repercussões como dispareunia, IST, gestações não planejadas, sofrimento psíquico e a sensação de desconforto com o próprio corpo, este último em virtude da depreciação por parte dos cônjuges. Ao cederem ao sexo, as mulheres imprimem sentimento de repulsa e criam subterfúgios para evitá-lo, vivência que tem desencadeado bloqueios para novas relações.

Tais experiências mobilizam as mulheres a buscarem autoconhecimento e o auto prazer, lançando mão do toque corporal, inclusive da masturbação. A orientação dos profissionais de saúde no sentido de desmistificar tabus quanto a esta possibilidade se faz de grande valia visto que pode contribuir para o processo de (re)descoberta do prazer consigo e com o outro. Nesse sentido, chama atenção para o engajamento desses agentes frente à possibilidade de investigar as repercussões apresentadas pelas mulheres na sexualidade, suas causas e as possibilidades de avanços para restauração de uma vivência saudável da sexualidade.

Além disso, considerando as especificidades de acessar mulheres com história de violência conjugal, torna-se oportuno viabilizar espaços de interação entre elas e o serviço de saúde, a exemplo de grupos reflexivos, com o propósito de desmistificar tabus referentes a subserviência feminina no relacionamento, a qual historicamente orienta a mulher a voltar seus esforços apenas para garantir o prazer masculino. Com isso, a criação de espaços de diálogo que subsidiem o empoderamento feminino quanto à liberdade de exercer sua sexualidade centrada no prazer e na perspectiva de autossatisfação sexual é importante no processo de transformações dos comportamentos sociais e sexuais. Essas práticas poderão contribuir para prevenir as repercussões negativas relacionadas ao exercício da sexualidade e promover a qualidade de vida e saúde para as mulheres.

## Data Availability

Os dados de pesquisa estão disponíveis no corpo do artigo.
